# Extracts

**Published:** 1882-02

**Authors:** 


### Extracts.

MENSTRUATION AND ITS DERANGEMENTS.—*Menorrhagia.* —This term must be carefully distinguished from metrorrhagia, a task rendered easy by reason of the essential difference between the conditions which give rise to one and the other form of discharge. The former appears only at the regular periods at which the menstrual flow is wont to recur, whereas the latter is constantly present in patients who suffer from it. The causes tending to the production of menorrhagia are to be found in such excessive changes in the ovary as bring about a state of hyperstimulation of the organ, and these changes may be initiated either through the nervous, or through the vascular system, or as a consequence of constitutional state. In the first case, excessive activity of the ovary results in an increase of function in the parts connected by nervous communication with it, and in these circumstances the blood supply will undergo variation commensurate with the changes set up. In such conditions of the general system as are observed in anæmia, on the other hand, fluid is easily transfused from the circulatory organs, and goes to increase the natural discharge in menorrhagic patients. In such cases constitutional treatment, by iron and other appropriate remedies, must be encouraged; but there will be also encountered varieties of this class of cases, each demanding special treatment, the nature of which must be decided on the merits of each as it presents. In this connection it may be urged that no drugs are, strictly speaking, definable as emmenagogues. Some substances which, under certain circumstances, act thus, may, at another time, and under varied conditions, ensure quite opposite effects, and therefore it should be regarded as an improper license of language to retain the term emmenagogue as fixed and invariable for any particular remedy.

*Menorrhagia* may be of different types. The *congestive* variety is encountered in florid women, and where it exists the uterus will be found thickened, deeply injected, and from it will flow a blueish sanguineous discharge following the introduction of a sound. This condition will, moreover, be attended by dragging pains in the back, aggravated at the periods of menstrual flow, and which may be set down as due to a passive uterine hyperæmia. The indications for treatment of such cases point to relieving the local congestion, for which purpose leeching is probably most suitable; and it may be repeated when required. General treatment is also called for, and particularly should the liver be attended to, since it is generally found to be at fault; likewise the digestive organs. The drugs to be looked to for benefit are such as ergot, nux, etc.

In ovarian menorrhagia the symptoms encountered present well-marked features to indicate the source whence they originate. There are no signs to indicate that the uterus is directly concerned in their production; pain is felt in the groins and down the thighs, and may also be experienced in the mammæ, all these special signs of ovarian implication being absent in the form of menorrhagia previously described. As in it, however, the symptoms are aggravated at the menstrual period, and find an explanation in the physiological principles already enumerated. In these cases examination of the uterus reveals no abnormality connected with that viscus, but, on the contrary, pain and tenderness are always discoverable about the region of the ovaries. and at the same time possibly enlargement of the ovary itself. Also in these cases of true menorrhagia, the menstrual act occurs more frequently than it would under normal conditions of health, and this also serves to separate these cases from those of the uterine class, in which no such increased frequency is met with. The reasons for this peculiarity again are to be found in the structural arrangements and physiological influences at work in the parts affected, and a due understanding of them will determine the nature of the treatment best to be adopted. The guide to this is in the pathology of the state, and would lead to the neglect of astringent remedies, which, from their mode of action, are improper and unscientific, since attention is to be devoted immediately to the ovaries, the seat of the morbid process that is to be attacked. The most powerful agent that can be employed in this connection is undoubtedly bromide of potassium. It exerts a direct and considerable action on the ovary, inducing atrophic changes in it, and thereby affects speedy diminution in the amount of discharge set up by over-excitement in its tissues. The bromide exerts specific influence over the nerves supplying the ovary, and hence the importance attaching to its use in all those instances where these nerves are in a condition of undue stimulation. Other treatment must of course be determined on the merits of each case; thus iron may be beneficially prescribed, and especially the application of anodynes locally in the form of pessaries. One of the most useful formulæ of this description is, Dr. Meadows explained, the following: coneia, 1 gr.; and atropine, 1-12 gr., in a pessary.

Menorrhagia consequent on organic changes in the uterus, may be considered as being amenable to treatment, except in those cases where such changes are due to presence of a malignant growth. The commonest form of such organic changes is so-called "sub-involution" of the uterus. This, the opposite condition to "involution," is really understood when the sequelæ to delivery are considered. Following parturition the uterus is left in a condition of enormous hypertrophy, which however undergoes natural reduction, and in from three to four weeks in ordinary circumstances the organ resumes very nearly its natural condition. The process whereby this is effected is one of fatty degeneration, the uterine muscles, increase in which is that which produces the great enlargement of the organ, being thereby gradually diminished by resorption of the hypertrophied mass. The contraction of the muscular fibres themselves is a material operation, moreover, in bringing about the result; and anything which may interfere with due performance of such action will necessarily arrest the process of normal involution of the uterus, and set up a state of sub-involution, thus favoring the menorrhagic condition; and it is a matter of observation that this consequence of its existence may be present with greater frequency after abortion than when delivery takes place at the full term. In such cases, too, metrorrhagia will not rarely be discovered. The occurrences which mark this state of affairs clearly show how the discharge may be maintained during their existence; they include descent of the uterus, a consequence of its increased weight acting on the weakened supporting structures, and the increased tissue substance at the same time constitutes a much thickened wall, while the cavity of the organ may be even double its usual length. The presence of these conditions, taken together with the history of the case, enables a positive diagnosis to be immediately arrived at. Treatment is then comparatively simple. The indications are to relieve the atony, which is the most important depraved condition, and thus restore the structures to a state in which the arrested process may once more be pursued to the natural termination.

In those instances, however, where the history given is that of a chronically inflamed hypertrophied uterus, the treatment resorted to will be different, inasmuch as the operating causes are shown to be other than in the variety just considered. In order to meet and arrest menorrhagia due to sub-involution, attempts are directed to inducing tonicity and contraction in the uterus, for which purpose such remedies as ergot, cinnamon, nux, quinia, etc., will be found serviceable. Locally, the application of galvanism, if persisted in with a determination to overcome the menorrhagia, will sooner or later be attended with relief of the symptoms, and is well worthy of most careful trial.

*Intra-uterine growths* may be either malignant or non-malignant in character, and the symptoms of their presence may include both menorrhagia and metrorrhagia. The nature of the discharges, however, may undergo very considerable alteration in accordance with changes taking place in the uterus itself. It should be carefully remembered, also, that over frequent ovulation will give rise to excessive secretion, the discharge of which may be mistaken for evidence of a menorrhagic flow. The phenomenon of periodicity, in all such cases, will serve to arouse suspicion as to their real nature, and the further influences exerted over the discharge by, and through the ovaries, will afford confirmatory proof concerning them.

When the fact that the uterus does contain a growth of some kind has been accurately established, then, if it be malignant in character, further decision respecting its special nature will be materially assisted by the signs its presence affords. Thus, the situation and character of the pain experienced by the patient will be a valuable means of diagnosis in cancer of the cervix, scirrhus. Absence of painful sensations is by no means of uncommon occurrence, whereas in that description of cancer which is found invading the fundus uteri agonizing pain is an all but invariable accompaniment, and it is never unattended by severe attacks of pain. The knowledge already gained of the nerve distribution of the uterus will render the explanation of this difference a simple matter, as also it will indicate why operation on the neck of the womb may be resorted to without the fear of unduly disturbing the patient.

There is also a distinct connection between the pain experienced and the amount of discharge from the organ. Thus the greater the degree of pain the smaller is the flow; and the more considerable the discharge the less excruciating is the pain, or the greater, in other words, the freedom from pain. This correlation is of immense value as a means of diagnosis in case of cancer uteri, and will often become a guide in the question of operative procedure. Whenever a copious discharge is met with in patients who suffer but little, and in whom the presence of a cancerous growth has been made out, then the conclusion is justified that the cervix is the part implicated, and never the fundus of the uterus. Again, as to the appearance of the discharge. This will be of a dirty fœtid nature, only in cases of malignant tumors, unless it be that it arises from a sloughing sore, or from pent-up secretions.

The presence and nature of the growths under consideration need to be made out with care and certainty ere their treatment can be hopefully resorted to; and for this purpose several modes of investigating them must be pursued. Several aids to physical examination can be employed, and the chief may be noticed in succession. The fingers of the operator are the most important of all these aids; by them the size, consistence, etc., of the cervix are at once determined; they unerringly ascertain the presence of growths on the walls they are in contact with, and as well, too, they will enable a determination to be arrived at concerning the preservation of the tumor, whether that is it be properly associated with one or other of the groups of cancerous growths, or is of non-malignant origin, as, *e. g.*, polypi, etc.

At this point Dr. Meadows proceeded to describe at some considerable length the numerous tumors to be found in the uterus under abnormal conditions, and the way in which they would be found productive of menorrhagia. From this subject he then passed to a consideration of the second most important aid to physical examination, the *uterine sound*, which he likened to an elongated finger, by the employment of which valuable information might be gained concerning the length and extent of the uterine cavity, the situation and nature of growths in its walls, etc., etc. He next enumerated the various kinds of tents, sponge, tangle, etc , also of service as aids to examination, which acted by dilating the canal of the cervix, and so admitting freer inspection of the interior of the womb.

In respect of *interstitial tumors* of the uterus, Dr. Meadows strongly inclined to the view that *enucleation* is the most appropriate treatment to adopt towards them ; and he expressed his conviction that in the future it would be universally employed. In the majority of cases the tumor is, he explained, so free of the proper tissue of the organ as to admit of being safely everted, and he had been surprised sometimes to find how thin the wall could be which permitted this to be done with absolute safety.—*Abstract of Harveian Lectures by Alfred Meadows, M.D., F.R.C.P., in Medical Press and Circular.*

---

WHY WE COUGH AND HOW WE COUGH.—Let us see what a cough is. It is a sudden and forcible expulsion of the air from the lungs, preceded by a temporary closure of the wind-pipe to give additional impulse to the current of air. The effect of these spasmodic expirations is the removal of whatever may have accumulated in the air-tubes, whether a foreign body from without, as when a particle of food finds its way into the wind-pipe, or an accumulation of mucus secreted by the air passages themselves.

Coughing is in part a voluntary act. We can cough whenever we wish to, but frequently we are compelled to cough when we don't wish to. Nerves are divided into two classes, sensory and motor nerves. The former carry intelligence to the brain; they report any disturbance on the frontier to headquarters. The motor nerves then carry back the commands of the general to act. You tickle a friend's ear with a straw, and his hand automatically proceeds to scratch the itching member. A tickling sensation is produced in the throat by any cause whatever; the brain then sends back orders to the muscles concerned to act so as to expel the intruder, in other words, to cough. And that is how we cough.

The source of the impression may be various. Frequently it is due to an irritation of the respiratory organs by foreign bodies, dust, and acrid vapors, admitted with the air in health, or to damp, cold air itself, if the organs are particularly sensitive, or to the presence of mucus, pus, or blood, in disease. Inflammation, from whatever cause, acts as a source of uneasiness.

There are, as we all know, many different kinds of cough. Thus, we have the dry cough, without expectoration, and the moist cough, with expectoration. We have the short, hacking cough, resulting from slight irritation, and the violent, spasmodic, and convulsive cough, caused by a greater degree of irritation or some peculiar modification thereof. Then there are the occasional, the incessant, and the paroxysmal coughs, terms that explain themselves. Hoarse, wheezing, barking, and shrill coughs, are due to the tension or capacity of the rim of the wind-pipe, or other portion of the tube. The hollow cough owes its peculiar sound to resonance in the enlarged tubes or the cavities in the lungs, if such exist. Sometimes the exciting cause of a cough lies not in the lungs and respiratory organs, but in the stomach, liver, or intestines. In other cases there seems to be no real cause; it is purely nervous or hysterical.

Cough remedies should be suited to the kind of cough in question, and attempt, if possible, to remove the cause. It is evident that a cough may be lessened either by removing the source of irritation, or by diminishing the excitability of the nervous mechanism through which it works. Both methods are generally employed, and most of the popular cough medicines consist of an expectorant and a sedative, in some mucilaginous or saccharine menstruum. Sedatives lessen the excitability of the nerve centre through which the act of coughing is produced. Opium in sufficient quantities will stop any cough, but if the secretion goes on accumulating, the patient must be allowed to cough, or he dies of suffocation.

Glutinous and saccharine substances lessen irritation, and as it frequently happens that much of the irritation which occasions the cough exists at the root of the tongue, and in portions of the throat which can be reached by troches and lozenges slowly dissolved in the mouth; hence these often afford relief, especially in dry, hacking coughs and the so-called tickling in the throat. Iceland moss, marshmallow, and gum arabic belong to this class. Their power is probably due to their covering the inflamed and irritable surface directly with a mucilaginous coat, and thus protecting it from the action of the air and other irritants.

All coughing beyond what is absolutely necessary for the removal of the accumulated mucus should be avoided, because it injures the parts affected by friction, and because it exhausts the patient; for the muscular exertion employed in a violent fit of coughing is very considerable, indeed, the muscular effort exerted by a patient with a bad cough during the twenty-four hours is really more than equivalent to that of many a man in a day's work. Both sedatives and mucilaginous substances can be employed, then, to check the excessive amount of coughing over and above that required to relieve the lungs and bronchial tubes of their accumulated mucus. To facilitate the accumulation of this, expectorants of various kinds are administered, according to the necessities of the case.—*Boston Journal of Chemistry.*

---

QUININE IN GYNECIC AND OBSTETRIC PRACTICE.—*Conclusions.* –First. That an exalted reflex excitability of the cerebro-spinal centres, as well as general plethora, may be recognized as a characteristic condition of the pregnant woman from the date of conception to the completion of involution. They may be termed "the gravid development and exaltation of the nerve-centres."

SECOND. That this provisionally increased development and polarity, intended for the purpose of fetal and uterine growth, renders the woman, during its continuance, eminently liable to become the subject of various morbid reflexes more or less peculiar to her condition.

THIRD. That these morbid reflexes are of two perfectly distinct and dissimilar kinds, differing widely as they may happen to occur, before or after parturition.

FOURTH. During the entire period of pregnancy, and until after labor, the reflexes are of an *excito-motory* character, restricted to the muscular apparatuses of the uterus and of general volition. They are apyretic and non-inflammatory. Their paroxysms threaten premature expulsion of the fetus in pregnancy, and eclamptic convulsions in labor.

FIFTH. After parturition, the reflexes are of an *excito-secretory* character. They are propagated, through the ganglionic or vaso-motor nerves, to the blood-vessels and capillaries of the pelvic organs and tissues and of the general system. They are marked by fever, congestion, and inflammation, with their products and consequences. Septic fever and peritonitis, with arrest of involution and mammary abscess, are their not uncommon results.

SIXTH. That quinine, by its contractile action on the capillaries of the cerebro-spinal centres, exsanguinates their nervous structure and, more than any known agent, depresses the reflex excitability from which the varied morbid phenomena of both pregnancy and child-bed originate.

SEVENTH. That quinine, except in cases of idiosyncrasy, or from an injudicious administration of the agent, exercises no influence whatever to superinduce premature explusion of the fetus.

EIGHTH. That moderate cinchonism adjusted to the type and approach of the paroxysmal neuroses, which endanger the welfare of the fetus during pregnancy, is one of our most efficient resources in many cases of threatened abortion and of premature labor. During parturition it may give "steadiness" to irregular uterine contractions; and, continued during labor, cinchonism is, in a most valuable degree, prophylactic against threatened eclampsia.

NINTH. That the reflexes of child-bed pertaining as they do, primarily and principally, to the recently evacueted uterus—well likened to an organ in a traumatic condition—opportune and ready for the awakening of fever and inflammation, are of the greatest character, frequently tending to disorganization and death, or else to permanent and irreparable injury. These "reflexes" constitute a dreaded class of diseases, most commonly called "puerperal," which, by universal consent, must be *prevented* rather than trusted to efforts, so often unavailing, for their cure. To this end, the most valuable and reliable prophylactic method will be found to consist in *the daily administration of quinine to the degree of moderate cinchonism* from the day of parturition and to be continued daily until normal involution is safely secured. By the observance of this "routine" as a rule it is believed that the occurrence of puerperal diseases will be largely prevented, and that the rate of child-bed mortality will be greatly diminished.

TENTH. That cinchonism in its quality of preventing and controlling inflammation, whether traumatic or idiopathic, and of suppressing suppuration—all of which is due to its power over reflex excitability of the cord and its action on the capillaries—has a claim to antiseptic value superior to Listerism, and is less to be dispensed with than carbolic acid or any of the means and appliances of the recognized "antiseptic method." In general surgery and especially in uterine surgery, as well as after parturition, the combination of carbolized irrigations and applications to diminish *peripheral* excitability with persistent *cinchonism* to depress *centric* excitability should constitute hereafter an antiseptic method more reliable, generally practicable, and less to be dispensed with than the most faithful observance of the complex Listerian process.—*Henry F. Campbell, M.D., in Gynecological Transactions*, 1880.

---

CHRONIC RETENTION OF URINE IN ELDERLY MEN.—Chronic retention of urine is a slow and insidious process, by which the bladder often becomes gradually over-distended without the patient's being aware of the fact. This retention may not become complete for weeks or months, or it may always remain incomplete.

In chronic retention there is always loss of power of contraction of bladder with impaired sensibility.

Chronic retention arises—

1. From gradual, progressive, but often incomplete closure of the urethro-vesical orifice by an enlarged prostate.

2. From atony of the bladder which has not been the outcome of prostatic enlargement, but of a urethro-vesical muscular valvule, of a urethral stricture, of a stone, or of tumors of the bladder, etc.

3, From neglected acute retention.

4. It also results from such disease or injury of either of the great nervous centres as may give rise to loss or impairment of innervation of the hollow organs. In this last named class of cases, there is not at first atony but true paralysis of the bladder, and it is important to distinguish it from the others.

In chronic retention the partial obstruction of the urethro-vesical orifice proceeds from gradual encroachment by either lateral lobe or by the increasing median portion of the prostate.

In chronic retention there is usually decided prostatic hypertrophy, either of one of the lateral lobes or of the median portion; except, of course, in those cases of atony from a muscular valvule, from stone, from stricture, etc., or of real paralysis of the bladder.

Chronic retention does not generally arise from exposure to cold, etc., though it is sometimes the outcome of acute retention.

Chronic retention is not always immediately preceded by dysury and strangury, as both may have begun weeks and months previously, and it is not accompanied by violent straining. Often there are no rational signs of its existence.

Chronic retention is for a long time incomplete—the urine being voided *voluntarily*, but in a small, weak stream which, every few seconds, is suddenly interrupted, then succeeded by twenty or thirty drops. This may be continued to the last, or the retention may be complete after weeks or months, to be finally incomplete with involuntary dribbling which at first occurs only at night or during sleep.

In chronic retention the bladder is soft, slack, yielding, painless, and cannot be mapped out. Its palpation gives the idea of its being a flaccid bag containing a fluid by which it is not distended—often leading the inexperienced to think that the fluid is in the peritoneal cavity and not in the bladder.

Chronic retention is always accompanied by cystitis.

When a patient, who is tormented by frequent but incomplete acts of urination, obstinately refuses to seek surgical aid, his case progresses somewhat as follows: His sleep is greatly disturbed, either by these constant desires to urinate, or by the dribbling urine; his appetite fails him, and all his other functions are soon more or less impaired. Irritative fever supervenes from the existing cystitis and the steadily accumulating purulent, slimy urine. He has dull lumbar pains from distension of the ureters and renal pelves; his bowels are constipated and tympanitic; his tongue is parched, his speech is incoherent, his skin is dry (except toward the last, when it may be covered with cold sweat, partly as a result of general relaxation, partly as a final effort of nature to eliminate effete material.) Very little urine is now passing away, although the bladder is still full; the function of the kidneys becomes more and more impaired, until finally it ceases entirely. He then sinks into a state of muttering delirium, from which he is with difficulty roused, and dies from the combined effects of urinous, purulent and fecal intoxication.

It is not an uncommon thing for surgeons who have much to do with urinary diseases to be consulted by elderly patients, all of whom tell nearly the same story: "For several years past I have been variously treated for a urinary trouble, which has been to me and to my physicians quite obscure. I pass enough urine, and frequently too, I have been sounded for stone, but none found; I have no gravel in the urine, my kidneys are said to be sound, etc., bnt I am conscious that something is wrong. Will you, sir, tell me what is the matter and what can be done for me?" While the patient is speaking, everything is made ready; he is first required to urinate, and try his best to empty his bladder. The catheter is then introduced, and from two ounces to a pint or more of purulent stale urine is drawn off, confirming the diagnosis of chronic retention of urine.

The surgical treatment of chronic retention should consist (1) in withdrawing the offending urine, (2) in relieving the existing cystitis, and (3) in endeavoring to remove the obstacle to the spontaneous expulsion of the urine.

When the retention is due to stricture, successful evacuating catheterism, followed by the enlargement of the urethra—by dilatation, divulsion or urethrotomy—to or beyond its normal capacity, and by suitable irrigations of the bladder, will be sufficient, as a general rule, to cure the retention, the atony and the cystitis; but to prevent the recurrence, dilating catheterism will be required periodically (not less than once a week) for several years otherwise the stricture will re-contract and produce those first effects. I shall be equally brief with respect to the treatment of chronic retention arising from spasmodic contracture of the neck of the bladder or from a muscular valvule, and only say that the catheter should be used three or more times every day, to be followed by appropriate urethro-vesical irrigations. If these means fail, internal incision, or in exceptional cases excision, of the valvule will be indicated.

If, when chronic retention is the result of supramontanal hypertrophy of the prostate, it be discovered early, and ordinary skill and judgment be exercised in the case, it will progress quite satisfactorily for a great many years. In certain cases of complete obstruction, if the patient has been properly taught to catheterize himself, he will rely entirely upon the catheter for relief and never think of urinating spontaneously. I know several elderly gentlemen, now in good general health and quite active, who themselves have been using the catheter five and six times daily for the last five, ten and twenty years. But these successes are due to early beginning and to the greatest care in the use of the instrument, in spite of which an occasional attack of orchitis occurs.

First, let me express my own convictions as to the catheters, the use of which should in general be avoided. All metallic and very rigid non-metallic catheters, or soft catheters armed with stylets, should not be used under ordinary circumstances. If such instruments had never been employed to relieve retention of urine from prostatic enlargement, cases complicated with false passages, now so common, would be of extreme rarity. I have never owned what is usually called a silver prostatic catheter, nor have I ever seen a case which I could regard as requiring the use of such an instrument. The difficulties which arise from catheterism with metallic or rigid non-metallic instruments, are encountered, 1st, at or near the bulbo-membranous junction of the urethra from an inordinately deep sinus of the bulb, undue force causing the point of the catheter to perforate the mucous membrane and enter the spongy tissue, or plough its way between the membranous urethra and rectum, and sometimes even enter the intestine. This accident makes itself manifest by a more or less copious urethrorrhagia ; 2d, in the sinus of the prostate. This latter impediment is formed by a urethro-vesical barrier causing a deep pouch in the prostatic sinus. In this case, the same undue force may give rise to a longitudinal rent on one side or the other of the floor of the prostatic sinus, extending to or into the barrier, or else the point of the catheter penetrates and sometimes transfixes the barrier at its centre. Such serious injuries are not generally inflicted with soft non-metallic instruments, though when their eyes are improperly constructed they cause considerable irritation and even erosion of the mucous membrane. Therefore, one of the prime requisites of a good catheter is an eye as small as practicable, with perfectly smooth rounded edges. It should never have two eyes.

The catheter should seldom be under No. 9, which though it may be pliable, retains a certain degree of firmness, and can be much better managed. If it seems too large the surgeon should dilate the urethra.

Chronic retention of urine of a few months' standing is quite amenable to treatment, especially in well-nourished patients, who are otherwise in fair physical condition; but in those who, for two or more years, have been neglected, and whose general health is, in consequence, much impaired, the prognosis is ordinarily bad; for after this lapse of time the ureters and kidneys are irreparably damaged, and the most that can be done is to mitigate suffering.

When catheterism is supposed to be impracticable it has been the custom for many years past to puncture the bladder with an ordinary trocar, now an operation as unjustifiable as it is unsurgical. Mercier, after an experience of nearly half a century, says that he has never found it necessary to perform that operation, and regards it as belonging to the infancy of our art. I too, am happy to say that I have never practiced puncture of the bladder with the ordinary trocar. I have in some cases resorted to supra-pubic pnncture with the capillary trocar and used Dieulafoy's aspirator to pump out the urine, but after sufficient experience and close observation of the results of this operation, I have come to the conclusion that it is not as safe as it seemed to me at first, and should be reserved for cases of the greatest emergency, and in acute retention only—chronic retention has waited and can wait a little longer. I know of several deaths that have resulted from the escape, through the eye of the hollow needle during its withdrawal, of a drop or two of urine, which, entering the connective tissue, produced vast abscesses between the bladder and anterior abdominal walls, where, too, the greatest care had been observed throughout the whole process. This occurred in a case operated upon by myself, and I have no doubt that the same accident has happened to many others.—*J. W. S. Gouley, M.D., in Proceedings Kings Co. Med. Society.*

---

A Plea for Improved Vaccination.—The remedies for the existing imperfect protection against small-pox would, in my judgment, be found:

*First.*—In renewed attention to the selection of proper eighth-day lymph, more careful cultivation of bovine "virus," greater care in both cases to scrutinizing and tracing it to a pure source. Each supply should be tested on one healthy vaccinee before it is extensively inoculated. Especially is the precaution needed in the use of bovine "virus," which not rarely produces local irritation, whose effect is pernicious upon the protective influence afforded by it.

*Second.*—Too much attention cannot be given to the methods of vaccination now in vogue—the technique of this simple procedure. Those who are not familiar with the writings upon this subject by Marson and Seaton, would do well to study their valuable suggestions and elaborate directions, especially with reference to the watchfulness necessary when the vesicle is maturing or matured. The care, precision, and attentive regard for minutiæ which the men of Jenner's day and the time immediately succeeding bestowed upon this seemingly trivial work, may be profitably emulated by the present generation of medical men, if they would protect their fellow-men against the terrible scourge which now and then invades our cities and populous country districts.

*Third.*—A more thorough instruction of medical students in the details of obtaining supplies of vaccine material, in the methods of its propagation, in the technique of vaccination, in the diagnosis of the true from the spurious vaccine vesicle, is imperatively demanded. The medical student cannot be too deeply impressed with the idea that this operation, slight as it may seem, will confer greater benefits to the people under his care than all the skill he may acquire in surgery. The admirable instructions on vaccination, written by Mr. Simon, for guidance of "vaccinators under contract," by order of the Privy Council, July 20, 1871, are a model upon which teachers may successfully construct their lectures on this subject. No medical student should be allowed to graduate who does not exhibit a full appreciation of the importance of vaccination and a thorough knowledge of all its details.

*Fourth.*—I would suggest the inauguration of a new specialty. The "vacinator" would in our large cities (which are the habitat of most specialists) prove far more useful to the public than many of the specialists who now minister to its numerous "special ills." A vast deal of good would be accomplished by men who, like Marson and Seaton, have studied the subject of vaccination in all its details, and who would relieve the general practictioner of all care in this important matter. A large number of educated physicians may devote themselves to this branch of study and practice with credit and profit to themselves and to the profession. As it now stands, the general practitioner obtains but little thanks, frequently not even compensation (for it seems to be by many regarded as a complement to the labor case), for this work. The opprobrium, on the contrary, resulting from the insufficient protection due to various causes over which the busy doctor has but little control, is unstinting. The greatest benefit, however, would accrue from this departure. Just as in other departments vast results have been achieved by special study, so will in time be accumulated large stores of satistical information and practical improvements in the methods of cultivating the vaccine-lymph and transmitting the vaccine disease in its purity and genuine type. Let it be borne in mind that the grand aim of medicine, the "stamping out" of small-pox, has thus far not been reached. On the contrary, we are confronted daily by failures, and taunted by the frequent recurrence of small-pox epidemics. This failure of vaccination to accomplish its alleged mission is converting a credulous public to the doctrines of the anti-vaccinationists, and thus adding fuel to the disease. I have attempted to demonstrate the cause of failure and to point out the remedy. The immeasurable importance of this subject demands earnest and immediate action on the part of the medical profession.—*J. Baruch, M. D., in N. Y. Med. Record.*

---

Recent Progress in Gynæcology.—*Laceration of the Cervix as Compared with Complete Internal Division of the Cervix.*—In considering the extent of this lesion, which is followed by changes requiring, sooner or later, treatment by surgical interference, the author advances the statement which we have so often insisted upon, namely, it is only when the laceration is lateral and extends beyond the crown of the cervix upon one or both sides that evil consequences follow. Among the factors which derange the circulation of the uterus while undergoing involution, he gives prominence to open lacerated wounds of the cervix extending entirely through the cervical wall. He shows how the lips of the cervix are forced apart by the superincumbent pressure, the womb finally resting upon the inner surfaces of the everted lips. This gives rise to influences which bring about ulceration and areolar hyperplasia. No such conditions or influences are present in the division of the cervix, for then the uterine structure is healthy, the weight of the uterus is not increased, and the constant tendency of the divided surface is to unite by first intention. It is often with the greatest difficulty, in fact, that such union is prevented. The author concludes as follows:

I. Parturient laceration of the cervix occurs in a womb whose tissues are in a state of fatty degeneration, and therefore have no tendency to heal.

Division of the cervix is performed upon a womb whose tissues are in a perfectly normal state, with an almost insuperable tendency to heal.

II. Parturient laceration interferes with a physiological function of the womb, namely, the process of involution.

Division of the cervix interferes with no function of the womb, but on the contrary facilitates the performance of one of its most important functions.

III. Parturient laceration occurs at a time when the weight of the womb is so great as to force the divided lips apart by pressure against the posterior vaginal wall, and so prevent healing.

Division of the cervix is performed while the womb is normal in weight, and therefore the divided surfaces remain in apposition, and will heal as fast as they are allowed to by the physician.

IV. Clinical experience shows that parturient laceration is productive of a long train of distressing symptoms.

Clinical experience shows that division of the cervix produces no injurious effects if properly done; but, on the contrary, completely and permanently relieves a very painful and persistent disease.

*Inflammation of Skene's Ducts as a Cause of Vaginismus.*—Dr. Skene calls attention to a case in his practice where inflammation of the two small ducts on each side of the meatus urinarius was the cause of vaginismus, which yielded to simply slitting open the ducts from within outwards, and applying a bit of cotton wet with iron to prevent them from uniting.

*Removal of the Uterine Appendages.*—The author reports an oophorectomy. The patient was one for whom he had previously aspirated, by the vagina, an abscess of the right ovary, evidently occasioned by a sponge tent left for nine days in the uterine canal. Two years had elapsed since the aspiration, when, among other symptoms, the rise of temperature at night and the night sweats led him to suspect the presence of pus somewhere about the pelvis, although no fluctuation could be detected. An exploratory incision was therefore made through the abdomen, and the pelvis was found roofed over by adherent coils of intestines, which were lifted with much trouble. Below these the whole of the organs were matted together, and their identification was a matter of great difficulty. He finally succeeded in recognizing the right Fallopian tube, distended into a cyst, with greatly thickened walls, and full of pus. Below it and immediately adherent to it lay the ovary, as large as an orange, and containing some old cheesy matter, the remains, probably, of the abscess tapped more than two years before. The uterus was bound down in the cul-de-sac by old adhesions, and from these he relieved it. He found the left ovary adherent below the fundus, and from it the left Fallopian tube ran a circuituous course, like a sausage in appearance, and adherent to the pelvis, the uterus, and a piece of small intestine. It contained about two ounces of pus. Both ovaries and tubes were removed, the latter being cut off close to their uterine attachments. The hemorrhage during the operation was very troublesome, but was controlled by sponge pressure. The patient recovered without a bad symptom, and is perfectly free from pain for the first time since the incident of the sponge tent. The uterus is perfectly free, and any movement of it gives no pain.

If cases with so extensive adhesions can be successfully operated upon, it certainly opens a new field for the treatment of many of those hitherto regarded as most intractable.

*On Removal of Uterine Appendages for the Arrest of Uterine Hemorrhages*.—In a paper read before this Society, the author, Mr. Lawson Tait stated that his experience had shown that the removal of the ovaries was not a certain method of arresting menstruation, while removal of the tubes as well as the ovaries seemed to be so. He cited thirty one cases, twenty seven of which recovered from the operation and four resulted fatally. The following conclusions might, he thought, be legitimately drawn from them:

(1.) So far as its primary results are concerned, removal of the uterine appendages for the arrest of intractable uterine hemorrhage is an operation quite as easily justified as any of the major operations in surgery.

(2.) So far as its secondary results are yet known, it is an operation which yields abundant encouragement for its further trial.

As a conclusion indicated but not wholly proved, he thought he might formulate a statement that removal of both tubes and ovaries is necessary to the immediate arrest of menstruation.—*W. H. Baker, M.D., in Boston Med. Journal.*.

---

The Effect of Genital Irritation in the Production of Nervous Disorders.—We are apt to forget how easy it is to start a fashion in medicine. Let any one—if he be of eminence, so much the better—advance a new opinion, in support of which he shall cite cases that contain sufficient truth to give them an air of value at first sight, then let him make some earnest and confident assertions of opinion, he who deludes himself carrying, of course, infinitely more conviction than he who merely deludes others—and the thing is done. The journals hungering for novelties, circulate the news. The profession itself, I am tempted to say almost in proportion as it is intelligent and studious, has a strong tendency to mistake novelty for progress. From hospitals and clinics come manifold seeming confirmations. It may even happen that the original writer is outdone, and lengths are easily reached that were far beyond his vision. A warning voice may here and there be raised, but it must be of authority to create more than an echo, and even then it may fail to effect its purpose. So the discovery advances on a royal road, swelling with the bulk of success at every step. It is difficult to stay it. It is always difficult to dislodge an idea from the aggregrate mind of a mass of men, especially of professional men; but when it has stolen in under the guise of science or art it is likely to be guarded with a particularly jealous care.

The history of the question of reflex paralysis is an illustration of this course of events.

In this country the question has assumed a different phase during the last decade, owing to the energy and widespread reputation of a New York physician. In the cases of which we have been speaking the alleged cause of the paralysis was in the kidneys, bladder, prostate, urethra, uterus, or intestines. Dr. Lewis A. Sayre, in two articles, one published in 1870, the other in 1875, has claimed that phymosis, adherent prepuce, and irritable clitoris, are frequent causes of paralyses in childen, of retention of urine, and of many slighter nervous disorders. Dr. I. Baker Brown, of London, had before this set the medical world of England by the ears concerning the value of clitoridectomy for the relief of epilepsy, hysteria, catalepsy, etc., and had provoked a furious discussion, partly medical, partly ethical, that culminated in his trial before the Obstetrical Society of London, his expulsion therefrom, and his resignation from the London Medical Society—harsh measures, doubtless, which could only have been taken in consequence of grave indiscretion on the part of the accused, or because of great intolerance on the part of his accusers. Mr. Thomas Bryant, in 1868, had narrated the histories of eight cases of urinary difficulties in childhood from elongated and adherent prepuce. In 1872 Barwell followed with the narrative of two cases of paralysis in children claimed to have been relieved by circumcision. Dr. Otis, of New York, has supplemented Dr. Sayre's second paper with several interesting histories of nervous irritations thought to have been caused by this genital lesion. The matter has attracted widespread attention, so much so that one would be thought to be lacking in precision did he not examine the genitals in all cases of slight nervous disturbance. Some enthusiasts have carried the matter so far as almost to look upon circumcision as a panacea, and one well-meaning gentleman of my acquaintance, who had charge of a general clinic several years ago, made such slaughter among the innocents that the mothers of his section of the city ceased bringing their male children to him. Another gentleman, even, as we shall learn later, believes that he has relieved cerebral softening by circumcision, as he was courageous enough to say, ten years before, that he had cured a general paralysis with insanity in the same manner. A matter that to use Bacon's familiar phrase, thus "comes home to our businesses and bosoms," merits critical examination.

To sum up, there have been twenty cases in all reported of alleged paralysis from genital irritation. In not one is the proof conclusive of this relation between cause and effect.

I have taken the trouble to address letters to Drs. S. Weir Mitchell, J. M. Da Costa, A. Jacobi, J. S. Jewell, Robert T. Edes, F. T. Miles, S. G. Webber, E. C. Spitzka, E. C. Seguin, F. P. Kinnicutt, T. A. McBride, asking them if they had ever seen a case of paralysis from genital irritation, in which the proof of the facts was conclusive. They have all courteously answered me, and positively in the negative. Dr. Newton M. Shaffer has expressed the same opinion in a late article; and Dr. William A. Hammond has never seen a case resulting from preputial irritation.—*Landon Carter Gray, M.D., in Annals of Anatomy and Surgery.*

---

ANTISEPTICS AND DRAINAGE.—As regards the use of antiseptics in abdominal surgery, my present view is that we must admit that a practice based on the principles of securing as perfect cleanliness and as sanitary surroundings for the patient as possible, thus reducing to a minimum the introduction of putrefactive elements, and yet at the same time quite distinct from the special use of antiseptics proper, is attended with very great success: but, as the addition of special antiseptics, *e.g.*, the use of carbolic acid, has produced on the whole even better results than the first mentioned system, it would seem that we are really bound to give our patients the benefit of the improvement, greater or smaller as it may be, of the special antiseptic over the merely cleanly system.

Drainage is much less frequently necessary now than was formerly the case. If antiseptics are adopted it is quite certain that a considerable amount of serous and even sanguineous oozing may take place within the abdominal cavity, and yet the patient do well; though it must be borne in mind that the absorption of this effused fluid must entail an additional burden on the patient, and that it will be influenced to an extent at present uncertain, by the condition of the peritoneum as an absorbing surface, as well as also by the condition of the patient, weakly or otherwise.

A considerable number of lives have been, and will continue to be, saved by drainage. Marion Sims was fully alive to this when he, some years ago, advocated very frequent drainage through Douglas' space. His error then lay, not in the principle, but in the detail. He drained in the wrong place. It is now well known that there is a condition of acute septic poisoning, precisely similar to, and due to the same causes as that which Dr. Matthews Duncan has named in the puerperal patient as "sapræmia." It arises from the absorption of pent-up putrid fluid. In such cases the symptoms rapidly develop and are of the gravest character; whilst by the timely escape of the putrid blood or serum, they rapidly subside and the patient is saved. This is done by opening the lower end of the wound, and inserting a glass drainage tube, and retaining it until no more fluid can be obtained through it, at the same time keeping it thoroughly syringed out from time to time with a disinfecting fluid, such as diluted sulphurous acid. This solution is better for this purpose than carbolic acid, on account of the coagulating property of the latter, and it appears to be equally efficacious.

The use of carbolic acid as an antiseptic is not altogether unobjectionable, and probably, ere long, some other agent will supersede it, which will be found to possess its perfect antiseptic properties whilst its objectionable features are absent. With a few surgeons there has latterly been an exaggerated fear of the evil consequences, and of the frequency of carbolic acid poisoning. In about 150 abdominal sections I have not been able to clearly detect carbolic poisoning once; and in no case has the urine assumed the peculiar character.—*Abstract of Ingleby Lectures by Thomas Savage, M.D., M.R.C.P., in Birmingham Med. Review.*

---

A New Method of Trephining the Skull and other Bones.—A short time ago I became cognizant of the method used by Prof. James E. Garretson for the removal of the coccyx. This he effects by uncovering the bone and grinding it away with the Bonwill surgical engine armed with a burr. A few days later, by his invitation, I saw him remove in a similar manner the right superior maxilla, which was the seat of an antral exostosis. The delicacy of manipulation, the absence of facial scaring, and the undoubted power of the engine, combined to give me a very high appreciation of its possibilities. Especially was this the case because my experience some three years ago with the so-called dental engine was very unsatifactory in surgical operations on bone.

During Dr. Garretson's operation some one of the bystanders suggested to me that the engine might be used for trephining, and, as I had shortly before been teaching this operation to my class, I was struck with the idea. It has been heretofore suggested, I believe, that the engine might be employed to drive a trephine, and thus cut out a disk or button of bone.

My idea, however, was that, as the ordinary trephines are usually of too great diameter and cause larger openings than are required for the insertion of the elevator, it would be practicable to bore a small hole in the skull by using in the engine a burr cut or roughened on its flat extremity.

As no patient was at hand, I utilized a cadaver for the experimental demonstration, and fractured the skull by means of a hatchet. I found that the burr called by the dental instrument-makers a fissure-burr, and which has a cut face, answered admirably. I applied it to the sound bone at the edge of the depressed fracture, and found that I could quite readily make a circular cavity in the outer table. This was carefully deepened until the viterous table was perforated. As there was no disk to remove, and as the burr, which I kept moistened with water, dropping from a cloth, threw out all bone-dust, the depth and character of the perforation were readily watched. When the skull was thus pierced by a round orifice about one-quarter of an inch in diameter, the elevator was inserted and the depressed fragments elevated and, where loose, removed. Sharp and irregular edges were equally well trimmed smooth or cut away by the burr.

When the rapidly-rotating burr is placed in contact with soft tissues, as one's finger, it can be pressed upon with considerable firmness without abrading the surface, while osseous tissue is quickly ground away. Hence it seems as if the meninges of the brain might be touched by the burr without injury being inflicted at the time the vitreous table is perforated. In fact, I am inclined to believe that the dura mater would be pushed in front of the burr and remain practically uninjured. This can only be tested in living animals or human beings, because in the cadaver the brain does not entirely fill the cranial cavity, though the dura mater may remain attached to the inner table. The depressed fracture, moreover, usually pushes the dura mater downward, which would thus be likely to be torn off from the sound bone nearest the depression.—*John B. Roberts, M.D., in Philadelphia Med. Times.*

---

UMBILICAL HERNIA.—Another trouble often met with at or soon after birth is umbilical hernia. This is, I think, the most frequent form of rupture in infants. The cause is to be found in retarded development. The abdominal plates of the embryo growing toward each other to form by their union in front the abdominal cavity, may fail to come together at the umbilicus. Thus there may be at birth a space large enough to favor the escape of a knuckle of intestine. In this condition, although the liability to hernia is congenital, the protrusion may not actually take place until some days after birth, when the intestines, now filled by aliment, are crowded downward and forward by the contractions of the diaphragm and abdominal muscles in breathing and crying. In size the rupture varies. It may be as small as a marble or as large as an orange, though I have never seen one so large as that.

*Treatment.*—At first, until cicatrization at the navel is complete, a folded cloth placed under the bellyband may be trusted to keep the intestine back. Afterward you may resort to repressive measures which are more reliable.

A piece of cork or soft wood having been shaped to the size of the opening and covered with patent lint or soft leather, may be stitched to elastic bands that pass around the body to hold it in place. Or, if you would have a dressing as *recherche* as possible, you may order a flat rubber air-bag and secure it between two layers of elastic webbing, the ends of which are to be secured behind the back. The apparatus should be kept constantly applied. By degrees the abdominal walls close and reduce the size of the hernial opening. At the same time the intestine increases in volume and protrudes less readily.

The treatment may need to be continued for some months. Nature may be trusted to effect a cure if you will keep the intestines back. Indeed I think that in many instances nature, without aid, is adequate to the cure.

As I shall not again call your attention to the subject of hernia in infants, I will say here that of the other forms, inguinal and femoral, so often seen in adults, the latter is almost unknown to early life, while the former is more frequent, "Most inguinal herniæ," says Vogel, "disappear spontaneously without truss or bandage." This is most fortunate, since it is well-nigh impossible to make use of adequate repressive measures during the first year or two of life. Nor is it so necessary, as in grown people, to keep the hernia reduced, as strangulation almost never happens to infants. I think it is well to reduce the intestine every time the child falls asleep. As babies sleep so much, the rupture is thus kept back the greater part of the time with but little trouble and at no cost. Besides, in well-nourished infants, the increasing amount of fat in the abdominal walls and around the groins tends to keep the intestine within the abdomen. When the child is older—two or three years—if the hernia is not cured, you may apply a truss with some little hope of having it worn without constant displacement or troublesome abrasion.—*William T. Plant, M.D., in Louisville Med. News.*

---

HYPERIDROSIS OF THE FEET.—Hyperidrosis of the soles is a common affection, but the treatment is often difficult. We should, therefore, never be too confident about the success of any one remedy, as it is often annoying to find out that the expected relief does not come. The prognosis should also be guarded, as the disease is often very obstinate.

The treatment in this case will consist in the local application of lotions, which, I think, yield more satisfaction than ointments. One of our very best remedies is belladonna in the form of the tincture. It should not be used too strong at first, and in this case I will advise one teaspoonful in one ounce of water, increasing to full strength.

*Psoriasis.*—The diagnosis is easy. In the treatment the first thing is to employ means to free the skin from the scales which collect more or less rapidly upon the surface. I would, therefore, advise the patient to take a bath every day, remaining in the bath half an hour, and rubbing the parts well with sapo viridis—after which to anoint the affected parts freely with olive oil. Internally, I will prescribe ten drops of liquor potassæ, freely diluted after each meal.

The prognossis, should be guarded. It usually requires months to effect a complete cure, and relapses are very liable to occur.

*Eczema of the Anus.*—A man sixty years of age presents, as you see, a very angry-looking lesion about the anus. The parts exhibit a raw surface, much inflamed and thickened, of a brightened color, and covered with some fluid exudation. External hemorrhoids are also present. The subjective symptoms are almost constant and exceedingly annoying—such as burning and itching. They are worse at night, and are often so severe as to keep the patient awake. This patient states that he has been kept awake for several nights in succession, and that his general health is being underminded. As for treatment, I would first direct the part to be treated with black wash diluted one-half, after which zinc ointment, which should be kept up for three or four days. Later, a tarry ointment may be ordered.

The bowels should be kept on the verge of purgation for a week, after which, arsenious acid, grain one-thirtieth ; reduced iron, grain one ; will be prescribed.—*Louis A. Duhring, M.D., in American Specialist.*

A Case of Popliteal Aneurism Cured by Means of Esmarch's Bandage and Digital Compression.—E. Z. Derr, M.D., U. S. N., reports the following case: The patient, a strong, robust man, was admitted to the U. S. Naval Hospital, Norfolk, Va., on April 15, 1881. Examination revealed the existence of a large, pulsating tumor in the left polipteal space. The calf of the leg was very much larger than its fellow, and both sensation and motion were very much diminished. Owing to this condition of things, no active surgical interference was resorted to until June 10th, when Esmarch's bandage was applied, and allowed to remain on forty-five minutes. At the end of that time the pain became so intense that it was removed. Pulsation returned after the removal of the bandage, but its force was perceptibly diminished.

On June 16 another application of the bandage was made, and allowed to remain one hour and twenty-five minutes. Pulsation returned on the removal of the bandage, but its force was still further diminished.

On July 9 the bandage was again applied, and allowed to remain one hour. At the same time digital compression was made over the femoral, and continued for six hours after the removal of the bandage. Pulsation returned after the cessation of the pressure, but was finally diminished, and the tumor was found to be much smaller and firmer.

10th. Digital compression was continued for two hours, causing still further diminution in the size of the tumor.

11th. Compression was continued for three hours to-day, with further good results.

12th. The compression was kept up for five consecutive hours to-day, and on removal of pressure no pulsation could be detected.

At the present writing—September 9—a hard, fibrous mass, not larger than a walnut, occupies the site of the large, pulsating tumor which previously existed.

The case is reported because it furnished another illustration of the utility of the combined method in the treatment of popliteal aneurism.—*Medical Record.*

Asthma.—Apomorphia subcutaneously, in 1-10 grain doses, has been found effective.

| | | |
|---|---|---|
| ℞.—Lobeliæ folv. pulv. - - - | } | aa ʒij; 8.00 Gm.; |
| Stramonii fol. pulv. - - | } | |
| Belladonnæ fol. pulv. - - - | } | |
| Potass. nitrat. pulv.- - - - | | ʒiij; 12.00 Gm. |

M. Keep tightly corked.

The patient is required to shut himself in a small, warm, close room, and sprinkle the medicine on live coals, or smoke it in a fresh clay pipe, until relief is obtained, which is usually within ten or fifteen minutes.

Dr. R. M. Lawrence, who has systematically studied the effect of ethyl iodide, recommends inhalations of fifteen to twenty drops of this ether from a handkerchief, repeated three or four times daily. It keeps the system constantly impregnated with iodine, and proves a most useful agent in spasmodic and other forms of nervous dyspnea, as also in the dyspnea of chronic bronchitis. We may mention besides the good results obtained by its inhalation in hay-asthma. It seems to favor oxygenation of the blood and to stimulate the respiratory muscles.

| | |
|---|---|
| ℞.—Tinct. lobeliæ - - - | ℥j. 30.00 fl. Gm.; |
| Ammon. iodidi - - - | ʒij 8.00 Gm.; |
| Ammon. bromidi - - | ʒiij. 12 00 Gm.; |
| Syrup. tolutan. - - - | ℥iij. 90.00 fl.Gm. |

M. S. A tablespoonful every one, two, three or four hours. (Bartholow.)

Of this prescription Dr. Bartholow says, "It gives relief in a few minutes, and sometimes the relief is permanent.—*Prescriber's Memoranda.—San Francisco Western Lancet.*

---

A Word for Truth.—In a letter to the *New York Medical Record* from Dr. J. Crichton Brown, London, wherein Dr. Beard and some of his hypnotic experiments are sharply criticised, the following closing paragraph is found:

"It is an ungracious task to refute error and unmask deception, but it is a needful one in these days, and one peculiarly incumbent on those practitioners of medicine who love and honor their profession, and desire to secure for it a large measure of public usefulness and respect. In no way can the profession of medicine be more surely degraded than for those who follow it to exhibit a childish credulity or countenance the arts of the charlatan. Empiricism is the sin that doth most easily beset it, and it behooves the profession, therefore, jealously to guard itself against any teachings or practices that tend in that direction. That the teachings and practices of hypnotism may have that tendency will scarcely be gainsaid, and hence the duty imposed on the medical profession, to keep a vigilant eye on hypnotic manifestations, and to repudiate at once any sympathy or connection with the vagaries and cheats and quackeries, that are apt to gather round the small central nucleus of established hypnotic facts. Ample toleration must be shown to the widest diversities of honest and intelligent belief, but no quarter must be given to the delusions of the weak-minded, the whims of the superstitious or the unworthy pretensions of those who are in too great haste to grow rich."

---

A Perffct Solvent for Quinine Sulphate.—I was called to see a patient some time ago ill of remittent fever, who could not take pills or swallow quinine unless it was made in solution. I was put to thinking of some way to give it, and looking over my medicine case, the first article I noticed was a bottle of the sweet spirit of nitre, and to one ounce of this I added twenty grains of sulphate of quinia, and found, to my surprise, that I had discovered a perfect solvent for quinine sulphate. I use the spirit of nitre whenever I want a solvent, since it makes a beautiful solution, and appears to counteract, in some measure, the nausea which is so often troublesome in fevers. My reliance, however, to counteract nausea and headache in fevers is laid upon the potassium bromide. I generally leave about two drachms, directing the nurse to put it in a glass of water and allow the patient to drink small quantities through the day, and especially after each dose of the quinine. I have found this to relieve patients to a considerable extent, if not altogether, of sick stomach, ringing in the ears, etc.—*R. C. Keuner, in Louisville Med. News.—Va. Med. Monthly.*

Cyst of the Pancreas.—Dr. Nathan Bozeman presented a cyst of the pancreas, which had been mistaken before removal for ovarian cyst.

The patient was the wife of a physician of Texas, 41 years old, robust, and weighed 200 pounds. She had been perfectly healthy up to seven years ago, when she began to suffer from pain in the right inguinal region, hip and thigh, and her abdomen began to enlarge.

This gradually progressed up to six or seven months ago, when she was examined by an eminent physician of New Orleans, who diagnosed ovarian cyst. She was subsequently sent to me, and I also thought the tumor ovarian, and decided on operation. This was done December 2d. Instead of ovarian cyst I found the tumor attached to the pancreas at junction of outer one-third and inner two-thirds.

The patient had been prepared for the operation by administration of salicin 15 grs. t. i. d., and was thoroughly cinchonized the day of the operation.

The patient after operation progressed as satisfactorily as any case of ovariotomy. There had been no symptom of interference with the function of the pancreas before operation.—*Proceedings N. Y. Pathological Society, in Med. Gazette.*

---

Treatment of Acute Articular Rheumatism.—Dr. Carpani divides the methods of treating this affection into four groups: 1st, with salicylate of soda; 2d, with quinine; 3d, with benzonic acid; 4th, with the application of vesicants *loco dolenti.* He lays down tho following maxims: 1, the salicylate is of value in cases of acute febrile polyarthritis with marked articular manifestations, unless it be contraindicated by concomitant cardiac affections, nervous, gastro-intestinal, or renal troubles; 2, the bisulphate of quinine is to be employed when rheumatism is associated with or dependent upon malarial poisoning; 3, benzoic acid is efficacious in cases of acute febrile articular rheumatism, complicated by nephritis; 4, vesicants (Dechilli's and Davies' method) are certain to cure rheumatism affecting one or only a few joints.—*Annali Universali and Racog. Med.—Med. Record.*

JABORANDI.—The greatest value of pilocarpin appears to consist in its power of causing rapid elimination of effete material in cases of scarlatinal nephritis. It has been frequently administered in such cases, and with much benefit; and it appears likely that, if it be given with proper precaution, it may frequently be the means of saving life.

*Quebracho.*—First introduced by Penzoldt, it has been found to be a decided palliative in many cases of dyspnœa. It is especially valuable in the dyspnœa of emphysema and chronic bronchitis. In dyspnœa depending on valvular insufficiency, its value is questionable.—*James Stewart, M.D., in Canada Med. and Surg. Journal.*

---

NEW REMEDY FOR BALDNESS.—In cases of confirmed baldness the new remedy proposed is to remove the scalp, bit by bit, and substitute, by skin grafting, pieces of healthy scalp, taken from the heads of young persons. The success which has heretofore attended operations of this nature in cases of scalp wounds gives a promising outlook for this new mode of curing baldness; and perhaps the day is not far distant when the shining pates of our venerable fathers will bloom with the flowing locks of youth.—*Independent Practitioner.*

---

A SUCCESSFUL GASTROTOMY has been performed recently by Mr. Jones, of the Manchester Royal Infirmary, England. The patient, a girl, aged 21, had stricture of the œsophagus, caused by swallowing nitric acid two years before. Bougies had been used at intervals, but finally none could be passed. The operation was performed at two stages, according to Mr. Howse's plan. Everything worked successfully, and the patient, since the establishment of the fistula, has gained twenty pounds.—*N. Y. Med. Record.*

---

MALIGNANT GROWTHS.—Encephaloid and sarcomatous growths have been extirpated so thoroughly as to secure the patient against the return of any sign of malignant disease. Epitheliomata have yielded to excision, and to the destructive effects of caustics. Then, why will the profession not cease the absurd talk about the cancerous diathesis, and the inherited tendency to malignant growths.—*Med. Herald.*

